# A case report on zinc phosphide ingestion resulting to acute pancreatitis

**DOI:** 10.1016/j.amsu.2022.103859

**Published:** 2022-05-24

**Authors:** Rupa Bhandari, Krity Basnet

**Affiliations:** Intern, MBBS, Kathmandu Medical College and Teaching Hospital, Kathmandu, Nepal

**Keywords:** Rodenticide, Zinc compound, Phosphide

## Abstract

Zinc phosphide is commonly used as a rodenticide and often abused for intentional self harm. For years, Scientist believed that, It gets converted to phosphine gas by hydrolysis in acidic circumference of the stomach upon ingestion. It is absorbed into the bloodstream and can affect cardiovascular, respiratory, hepato-biliary hematological and gastrointestinal tract. It also results into electrolyte imbalance and ultimately leads to multi organ failure. We present a case of 46 year-old-male with acute pancreatitis due to zinc phosphide ingestion that was successfully treated conservatively. Acute pancreatitis is a rare complication of zinc phosphide poisoning. It is crucial to identify zinc phosphide as the cause of acute pancreatitis and rule out other factors (like alcohol, drugs, genetics) contributing to acute pancreatitis. Interestingly, patient presents with only mild symptoms of poisoning as metabolic acidosis and abdominal discomfort, acute pancreatitis was incidentally diagnosed through lab investigations and radiographs reports after 3–4 days of ongoing conservative treatment. Management was conservative for acute pancreatitis. This article provides a comprehensive review of management of zinc phosphide poisoning induced acute pancreatitis.

## Highlights

• Zinc Phosphide is commonly used rodenticide in developing countries, intentionally misused for self harm.
• Recognized as a significant cause of morbidity and mortality.
• Mechanism of action is unclear.
• Phosphine gas is formed in the stomach after oral intake is rapidly absorbed inhibition of C oxidase, mitochondrial morphology and oxidative respiration are impaired at a cellular level.
• It causes various metabolic and non metabolic effects, resulting in life threating complication.
• Neither an antidote nor a specific treatment for it.

## 1 Introduction

Zinc phosphide (Zn_2_P_3_) is a metallophosphide commonly used as a rodenticide, Commercial products are often available in dark grey powder or pellets is generally misused intentionally for suicidal purpose in developing countries,potentially causing multi-organ failure [1].

Routes of entry in the body can be by ingestion, inhalation or via skin [2]. Clinical presentation of zinc phosphide poisoning is due to liberation of phosphine (PH_3_) on contact with acidic content of the stomach [1].

Recently some queries have been raised because of the distinctive presentation after poisoning and new idea was formed of absorption and liberation of phosphine through luminal tract [1]. Causes of Toxic induced pancreatitis are ethyl alcohol, methyl alcohol, scorpion venom, organo-phosphorus insecticides, pyriminil (Vacor), pentavalent antimonial agents, zinc phosphide [3].

The most common clinical presentation in poisoned cases includes nausea, vomiting, abdominal pain, hypotension, metabolic acidosis, respiratory alkalosis and acute renal failure. In some cases, some rare complications can be seen such as acute pancreatitis, pulmonary edema, transient hyperglycemia, transient leucopenia and intravascular hemolysis [2].

There is no specific antidote, resulting in very high mortality, and the key to treatment is rapid decontamination and applying resuscitative measures [4].

## 2 Case presentation

We report a case of a 46-years old male who presented to Emergency department approximately after 4 hours of ingestion of about 25 g zinc phosphide.

The patient past medical history is insignifancant and there is no history of daily intake of alcohol.

At time of arrival, he was drowsy with Glasgow Coma Scale of 13 with pulse rate 92/min, blood pressure (BP) of 110/80 mmHg, respiratory rate 16/min, and temperature 101 °F and SpO_2_ 92% in room air. He presented with nausea and vomiting. The Arterial Blood Gas Analysis at time of arrival showed metabolic acidosis (pH-7.34, PCO_2_-30.5, HCO_3_- 16.4 mEq/L).

Per abdominal examination was normal and bowel sound were present. The general physical and neurological examinations were normal.

An intravenous access was done for conservative management and was monitored through Electoccardiogram, BP, and pulse oximetry. Electrolyte imbalance was managed with 2 pints of crystalloids solution in 24 hours.

Gastric lavage was done twice with 40 gm of charcoal for Decontamination.

During this period, his vitals were being monitored and were stable.

He was then shifted to Medicine high care for further treatment and was monitored with electrocardiogram (ECG), BP, and pulse oximetry and serial ABG was done twice initially for initial 2 days which showed slight metabolic acidosis which were reversed through alkalizing agents. After decontamination and medications, the pH and bicarbonate levels were stable, suggestive of improvement and showing he was not severely poisoned.

On his 3–4th day hospital stay he was found to be drowsy, febrile (101 °F to 103 °F), hypotensive, mild epigastric pain, and distension and abdomen discomfort. Ultrasonography of abdominal and pelvis and blood investigation were done initially for presenting complains. Ultrasound of Abdomen and pelvis showed interstitial edematous pancreas. His laboratory investigation showed Serum amylase of 852 units per liter, serum lipase 1162 units per liter. There was no other significant past medical history and other co morbidities.

He was evaluated and treated with intravenous fluid, antibiotics, proton pump inhibitors, analgesics. After 3 days, when his pain subsided and Liver function test were within normal limits he was planned for discharge. On discharge, his vitals were stable and were sent with conservative treatment and psychotherapy was suggested and asked to follow up with LFT reports after 1 weeks. The total hospital stay was about 5 days. After 2 weeks of discharge from hospital his LFT reports were normal.

## 3 Discussion

There is huge number of cases reported on zinc phosphide poisoning yearly in developing countries. Zinc phosphides have been used as rodenticide, commonly used for suicidal attempts in developing countries [2].

For many years, it was believed that clinical presentation of zinc phosphide poisoning was due to liberation of phosphine (PH_3_) on contact with acidic content of the stomach.^1^However, relatively long hiatus between ingestion of Zn_2_P_3_ and presentation of its systemic toxicity, and progression of acute liver failure could not be account by the current opinion. Hence, other innovative theory signified that phosphonium, an intermediate product will create and pass through the stomach, which then will reduce to produce PH_3_in the luminal tract [1].

A dose of 5 g of zinc phosphide is toxic and can cause death. In about 30minutes of ingestion Phosphides produce toxicity rapidly and within 6 hours of ingestion death may occur. In one series, 55% of deaths occurred within 12 hours of ingestion and 91% within 24 hour [5].

Decontamination is done through gastric lavage using charcoal via naso-gastric tube. Recently, some sources have proposed the idea of using coconut oil, castor oil and potassium permanganate [1,6]. Decontamination and Gastric lavage was done through charcoal in our case too.

The most common Clinical presentation of phosphide poisoning is profuse vomiting, abdominal pain, palpitation, dyspnoea and tachypnea. Phosphide causes circulatory failure resulting in congestion and edema of most organs particularly, in the lungs.

Other features include disseminated intravascular coagulation, metabolic acidosis, or mixed metabolic acidosis and respiratory alkalosis, acute renal failure and acute pancreatitis ultimately multi-organ failure [5]. Phosphide poisoning is also known to cause glycaemic derangement. Transient hyperglycemia that could rarely occur is possibly due to pancreatic involvement [7].

Our patient was drowsy and showed signs of abdominal discomfort, nausea and vomiting at presentation. He denied any use of alcohol and any other factor that could contribute to acute pancreatitis.

Phosphine is a nucleophile and is capable of inhibiting cellular enzymes involved in several metabolic processes giving rise to various disorders and failures in organs and systems [8].

Sarma et al. Have reported that zinc phosphide ingestion leads to acute pancreatitis [1].

The United Kingdom guidelines for diagnosis of acute pancreatitis includes (not mandatory) rise of amylase (or lipase if available) within 48 h of characteristic abdominal pain. A high level of blood sugar, low level of serum calcium, evidence of metabolic acidosis at the time of admission, and raised amylase and lipase levels sub observed in the literature available. Given the temporal relationship between ingestion and onset and the absence of any risk factors precluding pancreatitis in this patient, we believe it is reasonable to suggest a probable cause and effect relationship [8].

Various studies have shown that ZnP are potentially lethal. Gokdemir et al. stated that mortality rate doubles with patient with elevated liver enzymes after poisoning [6].

In our case, acute pancreatitis was incidental finding as patient developed abdominal pain and fever on 3 rd day of hospital admission and through USG and lab investigation which showed showed interstitial edematous pancreas andelevated liver enzyme Serum amylase of 852 units per liter, serum lipase 1162 units per liter respectively.

The hypothetical mechanism phosphide–induced pancreatitis is that, released phosphine gas results in interaction and inhibition of intracellular enzymes involved in metabolic processes, the chief enzyme being the cytochrome *c* oxidase resulting in the release of hydrogen peroxide, superoxide, and other free radicals. Such redox-active compounds are toxic to pancreatic β-cells [8].

Alternatively,by Bogle et al. Pancreatitis could have resulted from widespread cytokine release, acidosis, and probably ischemia [8].

The exact pathogenesis of poshphine -induced organ toxicity is not well defined in the literature, however appears to be due to hypoxic and, if recovery occurs, it is complete, without residual effects.

Steinberg and Tenner described that numerous causes leads to acute pancreatitis, resulting from a mild disease to multi-organ failure and sepsis; it has an obscure pathogenesis, few effective remedies, and an often unpredictable outcome. Toxin-induced acute pancreatitis is uncommon. There is only five toxins described as toxicological causes for acute pancreatitis, ethyl alcohol being the most common, accounting for 35% of cases [3].

A report published in 1980 implicated pyriminil (Vacor), a nitrosourea-derived rodenticide in its aetiology. In a recent report, Gasser et al.' described pancreatitis induced by pentavalent antimonial agents, treatment of leishmaniasis. Zinc phosphide should be included in the list of identifiable, remediable toxic causes of acute pancreatitis [3].

The treatment for ZnP poisoning is supportive and symptomatic. There is a controversy regarding use of activated charcoal, but it is recommended to use [2]. According to an article of 2014 [1], more aggressive gastrointestinal decontamination is necessary in patients with positive radiography. Our patient initially came to emergency with gastrointestinal symptoms (nausea and vomiting), decreased level of consciousness, and metabolic acidosis. We observed him and started conservative therapy. After 3 days, feature of acute pancreatitis were initiated. We continued supportive care until his condition improved.

## 4 Conclusion

We have a case of acute pancreatitis complicated by zinc phosphide poisoning, without any previous risk factors leading to pancreatitis. Preceding the acute pancreatitis there is an alleged history of intake of rodenticides. Toxic induced pancreatitis is rare, other causes of acute pancreatitis were ruled out through blood examinations and imaging techniques before confirming that it is due to phosphide. Toxics effects are due to release of oxides that are toxic to beta –pancreatic cells. Treatment is always decontamination and symptomatic.

This case report has been reported in line with the SCARE Criteria [9].

## Ethical approval

This is a case report that does not need an ethical approval.

## Sources of funding

No any funding sources available for this manuscript.

## Author contribution

Rupa Bhandari and Krity Basnet worked for literature review, discussion of the case report and revision of the case report into its final version. Krity Basnet took the relevant history, clinical examination, collected relevant investigations of the patient and wrote the report. Rupa Bhandari helped in the revision of the case report into its final version. She was directly involved in patient's management in the hospital.

## Registration of research studies

This is a case report that does not need to be registered.

## Guarantor

Dr. Rupa Bhandari.

## Consent

Written informed consent was obtained from the patient for publication of this case. A copy of written consent is available for review on request by the Editor-in-chief of this journal.

## Declaration of competing interest

No any conflicts of interest declared.
